# Building blocks of biofilms – an engaging and hands-on microbiology outreach activity for school children and the general public

**DOI:** 10.1099/acmi.0.000467.v3

**Published:** 2023-02-03

**Authors:** Hayley Pincott, Megan Hughes, Thomas Cummins, Daniel J. Morse

**Affiliations:** ^1^​ Show Me The Science, Cwmbran, UK; ^2^​ Oral Pathology & Microbiology, University Dental Hospital, Cardiff, UK

**Keywords:** biofilms, science communication, antimicrobials, infections, public engagement, microbiology

## Abstract

Biofilms are naturally occurring communities of micro-organisms, attached to a surface and often embedded in a matrix of self-produced polymeric substances. Biofilms are widely implicated in human infections, particularly on prostheses and medical implants. Such biofilms are difficult to eradicate, often leading to replacement of the prosthesis and resulting in a significant burden to healthcare. Here we present a fun and engaging interactive activity targeted toward primary school/early secondary school children, introducing the concept of natural and healthcare-associated biofilms, using dental plaque as an archetypal example. Dental plaque forms as a result of poor oral/dental hygiene, and develops according to a typical series of defined stages: attachment and adherence to the surface, followed by colonization and maturation of the biofilm structure, and eventually, dispersal. This activity uses dental disclosing tablets to visualize real biofilms (plaque) on the participants teeth, and uses interlocking building-blocks to represent microorganisms, where children build three-dimensional ‘biofilms’ of varying shapes and structural integrities. Each of the stages of development are discussed in detail, and after building the biofilms, balls of different shapes, sizes and weights can be used as ‘antimicrobials’ to disrupt the biofilm structure. The outcomes of the activity are to enhance knowledge and general understanding of biofilms; their ubiquitous presence in the natural environment, development, implications in healthcare, and challenges of treatment. The various ‘antimicrobial’ balls also provide a basis to introduce and discuss drug selection for infections, and the importance of using the correct antimicrobial for different infections to avoid development of resistance.

## Data Summary

Original data supporting analysis of impact are available at the following Open Science Framework DOI: 10.17605/OSF.IO/MDVPQ.

## Introduction

The need for promotion of STEM opportunities within education and careers has been a factor for many educational authorities in England and Wales [1]. This is of particular importance with the entirely revamped national curriculum for primary schools in Wales beginning in September 2022 [2]. Briefly, this new curriculum emphasizes thematic teaching practice rather than the traditional ringfenced subject-focused lessons. The ability for teaching staff to implement a range of traditional subjects within a theme will benefit younger learners who typically learn through play and experiences.

In addition to traditional teaching practices, external educational providers can enhance the learning for children in schools through provision of activities outside of but directly related to the scope of the day-to-day themed teaching. This outreach can supplement teaching practices with great success in terms of knowledge uptake and positive experiences [3]. Younger school pupils (early years/foundation phase) learn through play, which progresses to a more typical classroom environment as they grow. This natural transition prepares them for later educational experiences, such as secondary school and university. However, it does not mean the learning experiences through play and interactivity are not necessary or suitable for this primary school age group. Indeed, feedback we often receive from teachers and pupils alike indicate that learning uptake is higher for complex subject areas when learning through play and experience such as workshop activities, and particularly for perceived complex topics as in STEM subject areas.

Teachers can also benefit via professional development opportunities related to STEM outreach activities [4]. Often at primary school level, teaching staff are not subject experts, although they do have a very broad knowledge of many different subjects. Indeed, some subject areas are more difficult to teach than others, depending on the experiences of the staff member. This is particularly evident in STEM subjects, but partnerships with external STEM outreach providers has a mutual benefit for all stakeholders.

Furthermore, outreach/public engagement is certainly not limited to schools. The general public (not limited to children, but adults of all ages) benefit greatly from interactivity and novel experiences like STEM outreach. Lifelong learning is something that many strive for, and having positive experiences through novel interactive activities is one such way this can be achieved. Public festivals, science festivals, online STEM communities and pop-up events are some examples of ways that the general public can get involved with STEM regardless of the stage of their lives.

STEM outreach does not stop at purely theoretical knowledge or physical experiences, but the exposure to the diverse range of STEM professionals on a personal level. One of the first pre-workshop activities we ask school pupils to do is to describe (either verbally or drawing on a whiteboard) a scientist. Mead and Métraux [5] demonstrated that the majority of the time, people previously described a scientist as a white male, often with facial hair, and messy hair/appearance, glasses, and wearing a lab coat [5]. In our experiences at many schools and public events, this perception has not changed much in the past 65 years. This stereotype is not very conducive to enhancing diversity or highlighting the diversity or accessibility of STEM careers for anyone that does not fit that category. With an ever-increasing diverse population, this stereotype must change. Gender and ethnic diversity are key considerations when delivering outreach workshops, because to see people that children can relate to can have a profound effect on their self-belief, and perception of what careers are possible.

Capital comes in many forms, and intellectual and science capital are two important aspects for general successes in STEM [6, 7]. Much of this can be attributed to experiences in early life, through personal development, family dynamics and learning experiences. However, additional educational provision can enhance this science capital, also building on the learning through play approach of primary school teaching practices [8].

Complex scientific concepts and ideas often have a broad base of underpinning scientific theory. There are endless ideas of activities that can, and over time, will be developed to educate people on various concepts, and with our background expertise in healthcare and microbiology, we are focused on several key areas in these fields to promote the range of STEM education and career opportunities available. One of our flagship microbiology activities involves biofilms, with several key outputs detailed below.

Biofilms are commonly described as complex communities of micro-organisms, that attach to surfaces, and embed themselves in a self-produced enclosing matrix of polymeric substances including carbohydrates, proteins and extracellular DNA [9–11]. They are a common natural phenomenon and are the preferred mode of growth for many micro-organisms. One of the most easily identifiable and archetypal examples of a biofilm is dental plaque [12], with other important examples such as pond scum, biofilms on implanted devices and microbial fuel cells.

Biofilms develop according to a series of distinct sequential stages [12, 13]: starting with reversable attachment to the surface, followed by irreversible adherence and colonisation, maturation and matrix production where the biofilm grows as a three-dimensional structure, and finally (if necessary for the biofilm cells to survive) dispersal from the original biofilm aggregate to form biofilms elsewhere. This is particularly important in healthcare-associated biofilms, where secondary infections can occur due to dispersal of microbial cells from a primary biofilm. An example of this is endocarditis where micro-organisms from the mouth can enter the bloodstream and attach to the cell surfaces within the heart [14]. Biofilms are a significant healthcare burden, and their ubiquitous presence is often underappreciated by the general public.

Using the concept of biofilms as a natural phenomenon, we aim to highlight the presence and importance of microbial biofilms in everyday life, using dental plaque as the archetypal example, to which everybody is able to relate. The activity allows for the introduction of microbiology at a basic level: that micro-organisms come in many shapes and sizes (as do plastic building blocks); that they are ubiquitous in the natural environment (with the example of the oral cavity and saliva); and the mechanism of natural existence/persistence. Micro-organisms attach and stick to surfaces in a similar manner to that of building blocks, through ligand and receptor interactions, allowing them to persist in an otherwise difficult environment, particularly when considering the natural sloughing of cells, or salivary flow. The formation of biofilms then allows them to build a community, which for the benefit of the community as a whole, can be self-protective. Additional complex characteristics such as nutrient acquisition through pores and biofilm channels are introduced, and development of the physical biofilm structure. This has wide-ranging implications for general knowledge, especially around the theme of microbiology. Many children see ‘germs’ or microbes in a negative light, whereas in fact, they are typically harmless or beneficial for us as an organism. This is highlighted in the introduction of microbiology, demonstrating that without them, we would not survive ourselves! There are of course times where microbiology has negative effects on us as humans, and this is also discussed, as are medical advances such as medicines or treatments.

This activity serves to introduce biofilms as a concept, to explain their development and introduce medical and environmental treatments and highlight the ever-increasing threat of antimicrobial resistance.

Specifically, this activity was developed with several key objectives:

- to use an engaging and interactive activity to demonstrate the concept of biofilms, and the stages of formation, encouraging discussions of biofilm structural characteristics;
- to highlight the clinical importance and mechanisms underpinning biofilm formation;
- to demonstrate the inherent difficulty in physically destroying established biofilms using soft ‘antimicrobial balls’;
- to highlight the importance of correct and sustained medicine dosage.

The approach of this paper and developed activity is primarily from the perspective of dental plaque, but other suitable forms of biofilms including chronic wound biofilms or environmental biofilms can be used as alternatives.

## Procedure

### Practical activity

The intended audience for this activity includes school pupils of late primary (years 4–6, ages 9–11) or early secondary school age (years 7–9, ages 12–14). At public events, the activity can be adapted according to the ages of the participants, e.g. use of more technical vocabulary where suitable for adults, depending on competence. It is important to adapt the delivery to suit the audience. As the overarching intention of the workshop is to promote STEM, priority target audiences include schools in areas of higher socio-economic depravation, factors that indicate lower social status including levels of free school meals, or geographic areas that do not typically associate with later-stage STEM education, or STEM careers.

Ideally, group sizes of 10–12 for this activity would be suitable. The group is split into two sub-groups, and to ensure access and engagement of all participants, it is better to keep this to less than six per build. Additional base plates and building blocks can be provided to accommodate larger group sizes.

Two areas on one table were setup ready for the activity. These include (in duplicate) one plastic building block base plate, approximately 100 plastic building blocks of various shapes (square, rectangle and taller thin blocks), a set of soft snowballs, and table tennis balls for use as ‘antimicrobial balls’.

**Fig. 1.**
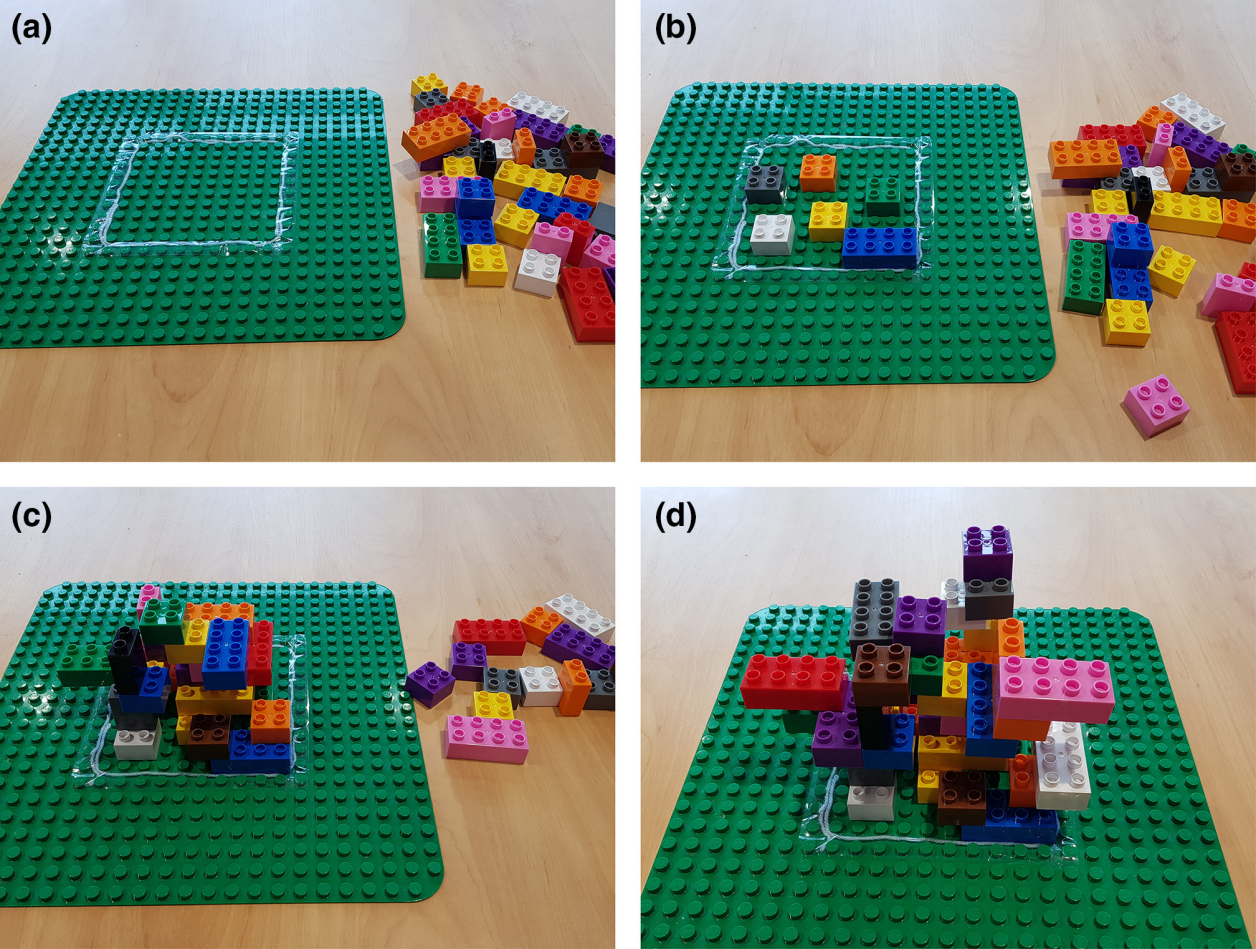
Biofilm formation occurs according to defined stages: (a) using the building block base plate and plastic building blocks, the first stage of biofilm formation is attachment (b) and adherence to a surface, followed by (c) colonization and maturation until grown into a mature 3D structure with channels (d).

A verbal introduction introduced biofilms by asking questions of the participants. Questions including ‘What is a biofilm?’, ‘Have you ever seen a biofilm before?’, ‘Where have you seen biofilms?’ were used to gauge knowledge, and then basic microbiology was introduced, including the types, shapes, sizes and locations of micro-organisms. Highlighting the diversity of micro-organisms in the natural and man-made worlds, giant microbe plush toys were used to emphasize the ubiquitous nature of micro-organisms. Using the example of the oral cavity, and asking further questions including ‘What happens if you don’t brush your teeth?’, and ‘How does it feel on the surface of your teeth if you don’t brush them?’, the different shapes and sizes of the building blocks, participants were shown that micro-organisms ‘float’ around in the saliva, where at close proximity to the building block surface, just as with the tooth surface, they are attracted to this surface through forces like van der Waals (electrostatic attraction). They then ‘stick’ to the surface, and if they are allowed to remain there without removal, stick very strongly through interactions between micro-organism and human cells (ligand–receptor locking interactions) (Fig. 1a). More building blocks were used to demonstrate the frequency and high rate of microbial attachment to the surface, and when the surface area was used to an extent where no other blocks can stick to the surface, the concept of secondary colonizers was introduced. Rather than stick to the surface, blocks then stick to other blocks, offset from a completely flush connection (important later) (Fig. 1b). This occurred over and over again, and a three-dimensional structure began to develop, Using more blocks, and sticking them to each other, a typical biofilm structure was achieved (Fig. 1c, d). This structure included channels for oxygen and nutrient transport to the inner depths of the biofilm and the micro-organisms at the base of the structure, as with actual biofilm development.

After the demonstration, the structure was dismantled, and the participants split into two groups. These groups then, within the rules of building in an allocated space, ensuring channels exist in the biofilm, and allowing connections between blocks but not entirely covering each block, were tasked with building a biofilm structure of their own. They were given 60 s to do so as a team.

After the elapsed build time, the biofilm structures were evaluated for structural integrity, shape, size and use of blocks. Then the teams were swapped over for the ‘treatment’ phase of the activity.

**Fig. 2.**
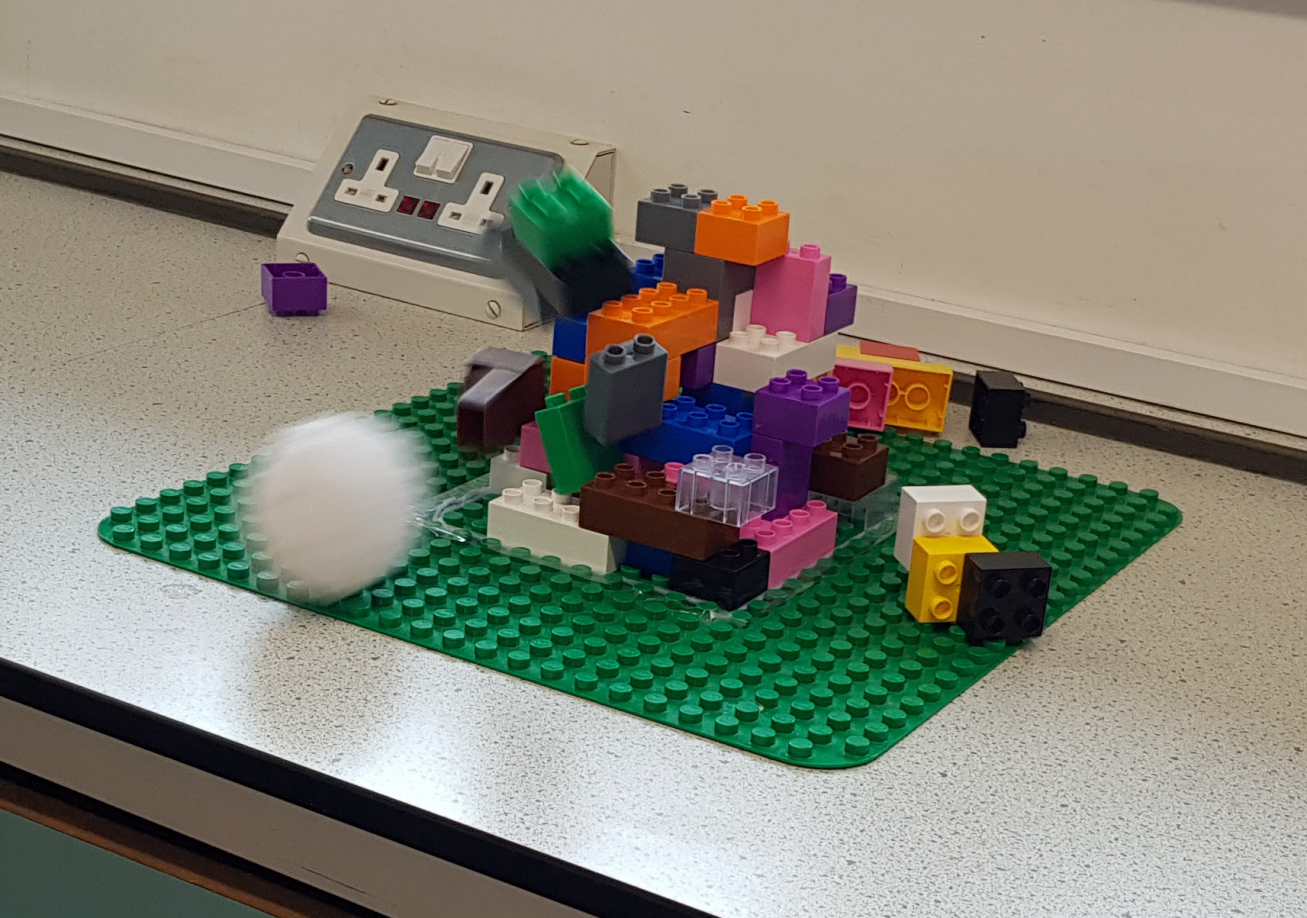
Balls that represent antimicrobials are thrown at the biofilm structure, to disrupt the biofilm as a treatment. The soft white ball has struck the biofilm, causing some structural damage and some blocks (representing microbial cells) to detach from the biofilm.

Giving examples of other biofilms, specifically those that form on medical prostheses (knee or hip replacement), the participants were asked how these biofilms could be treated, because unlike the oral cavity and dental plaque, these cannot be ‘brushed off’. The answer of ‘using medicine’ is common but can also be prompted by probing with further questions including ‘When you are unwell, who do you go to see?’, and ‘When you are unwell, what may you be given to help you get better?’. Use of medicines is quite structured, and is incorporated into this activity. Each participant was given one soft snowball, and use this to ‘treat’ or destroy the biofilm structure they have built (Fig. 2). After the first ball (first dose), the biofilm structure was evaluated, and typically remained quite structurally sound. This was then repeated for the second, third and fourth doses as necessary. After several ‘doses’ of medicine, the biofilm structure was re-evaluated, and only those that originally stuck to the surface remained. The participants were asked about these, and why they remained. Typical answers include ‘the medicine didn’t work’, or ‘we didn’t use enough of the medicine balls’. They were then asked to review the number of ‘doses’ used, and critically think about why it may or may not have worked to remove the biofilm entirely after the first dose, second dose etc. This highlights the importance of taking several doses of medication as necessary, and the right frequency. Further, the concept of ‘persister cells’ was introduced that show how some micro-organisms are either inherently tolerant to treatments or certain conditions, and those that may be resistant to treatment (through acquisition of antimicrobial resistance).

Use of table tennis balls was also used in a second biofilm build to emphasize the choice of the right medication for the right type of infection. In this case, they were given ‘antibiotics’ and told they were building a ‘fungal biofilm’.

Use of disclosing tablets (with drinking water available to rinse and waste collection cup) was also used to demonstrate real-life biofilm presence on the teeth of participants. This showed as coloured areas on the teeth, pink for relatively new established plaque biofilms, and purple for more established plaque. This was a very visual way to demonstrate biofilms in person, and emphasized the concepts we introduced in the earlier activity.

For evaluation, as the participants started with a known number of building blocks, they quantified the number of blocks remaining in their biofilm structure to determine which team has removed the most biofilm, and introduction of basic mathematical calculations such as percentage removed/remaining can be incorporated to increase educational reach. Adapting the biofilm structure, method, time allowed and resources available can be used to change the activity, and a short project based on height, structure strength or ‘antimicrobials’ used will also incorporate this.

### Data collection and evaluation

Evaluating impact for outreach activities is important, and this can be done in several ways. We have incorporated numerous methods of evaluating impact including token jar allocation for measuring self-certified enjoyment, keyword capture before and after the activity, numbers of participants that would consider a STEM career, and detailed feedback forms.

Our monitoring and evaluation programme is an ongoing task, where we continually review and evaluate activities. During recent school visits in the summer term of 2022 to primary schools we obtained the following data from participants (Table 1) and teachers (Table 2):

This data covers four primary school visits, with a total of approximately 220 pupil participants. Of the respondents (pupils *n*=120), on a scale of 1–5 (1 being strongly disagree, 5 strongly agree), the self-reported building blocks of biofilms scored as follows [average score (sd)]:

**Table 1.** Average scores of self-reported grades given to specific comments about the workshop, completed by participants

Comment	Average score (±sd)
The activity kept me interested most or all of the time	4.84 (0.51)
The activity was fun/enjoyable	4.79 (0.59)
The activity was suitable for my age/ability	4.82 (0.57)
The activity taught me something I didn’t know before	4.63 (0.87)

**Table 2.** Average scores of self-reported grades given to specific comments about the workshop, completed by teachers

Comment	Average score (±sd)
The activity was engaging/provided sufficient physical and mental stimulation	4.33 (0.58)
The activity was fun/enjoyable	4.67 (0.58)
The activity was suitable for the pupils age/ability	4.67 (0.58)
The activity provided new knowledge	4.33 (0.58)
The activity will benefit pupils in the short term (<12 mth)	4.33 (0.58)
The activity will benefit pupils in the long term (>12 mth)	4.33 (0.58)

The activity was delivered as part of a workshop with other interactive activities, and of a subset of 82 pupils, 43 chose building blocks of biofilms as their favourite activity.

Qualitative feedback from pupils included ‘building biofilms made science fun’

Teacher feedback indicated a similar trend of positive reception (teacher *n*=3).

Qualitative, free-text feedback on the question of ‘Were there any specific things you/they enjoyed?’ included

‘Introducing scientific concepts in a fun and engaging way’.

We measured enjoyment levels for each activity delivered at the workshop through tokens and a jar based on their feelings. We had a ‘smiley-face’ jar for a positive experience, and a ‘frowning face’ jar for a negative experience. We had 100 % positive feedback using this token method for the building biofilms' activity.

**Fig. 3.**
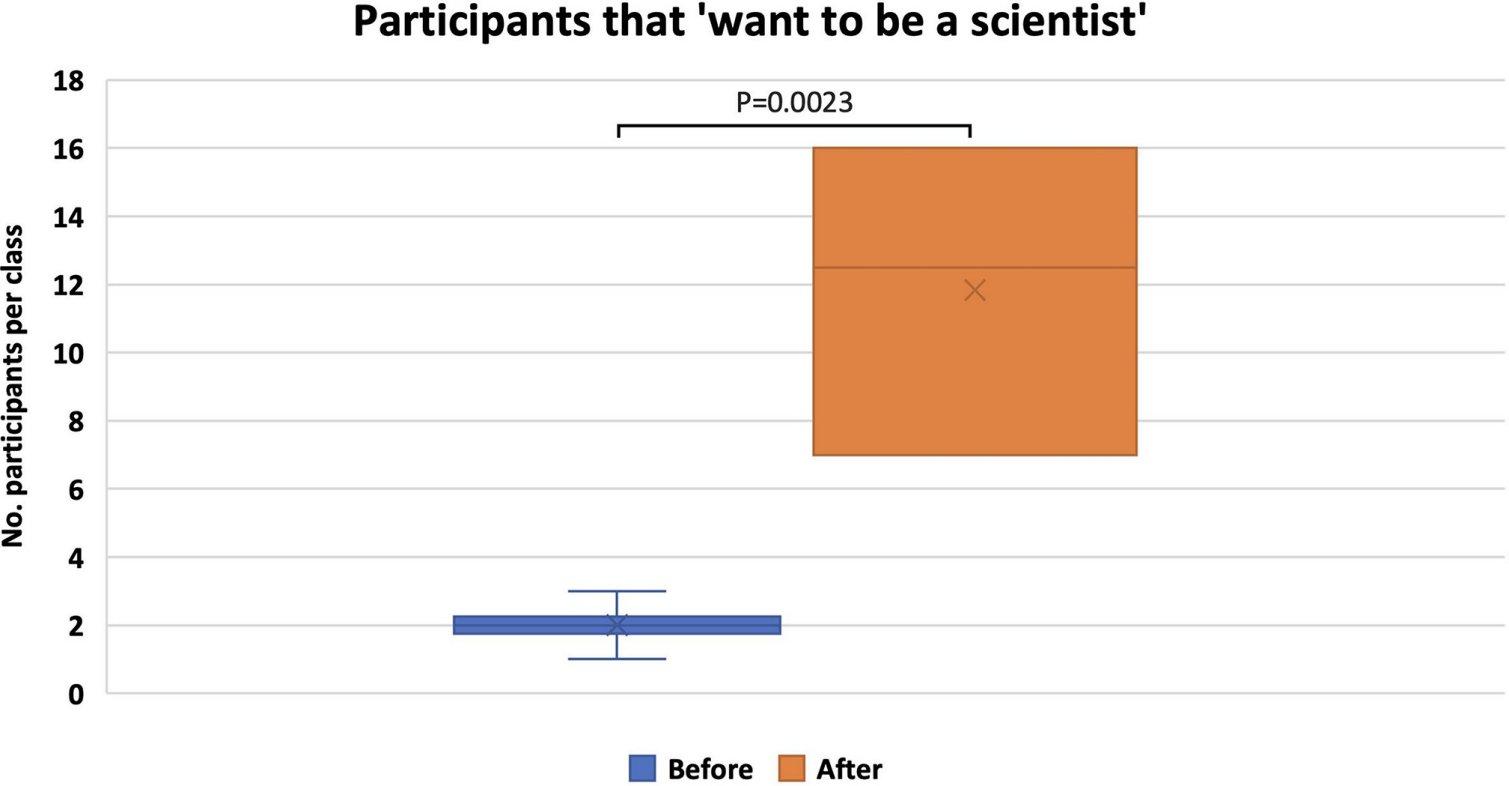
Box and whisker plot showing number of participants that ‘wanted to be a scientist’ before the workshop (blue) versus those that ‘wanted to be a scientist’ after completion of the workshop (orange). A significant (*P*=0.0023) increase in those that want to be a scientist was observed.

**Fig. 4.**
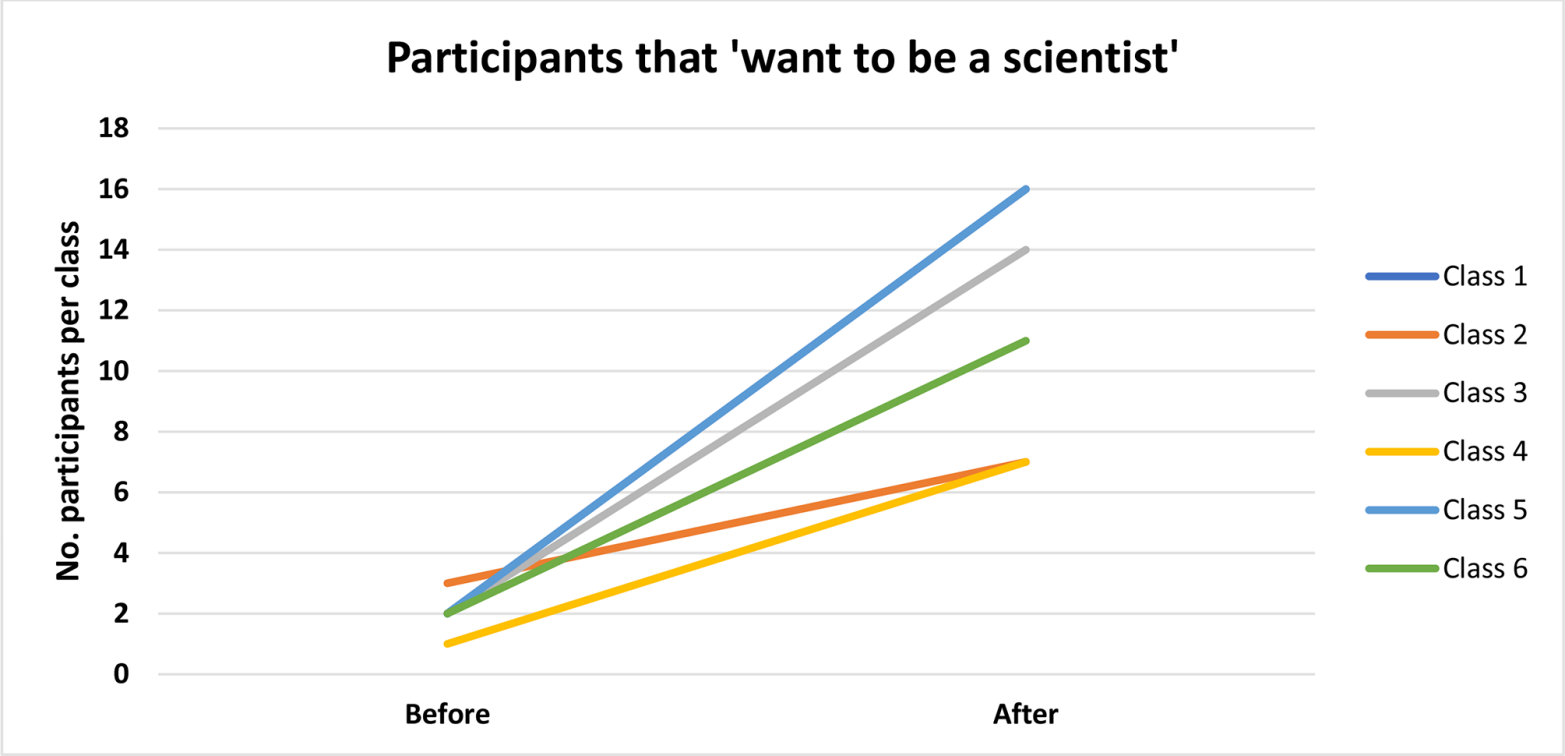
Increases ranging from 4 to 14 participants that ‘want to be a scientist’ were observed for individual school classes as a result of the workshop.

One of the key outputs we monitor includes self-declaration of whether participants would consider a STEM career after the workshop compared with numbers before (Figs 3 and 4) . During three school visits (*n*=6 classes), those that would consider a STEM career before the visit was an average of 2 (+/- 0.63), compared with 11.83 (+/- 4.17), an average increase of 9.8 participants per class. Statistical analysis of this data was completed using GraphPad QuickCalcs, using a paired *t*-test.

## Discussion

This activity was intended to introduce a very common, but technically quite complex phenomenon of biofilms. Using the example of dental plaque, a relatable example of biofilm formation, several concepts were introduced including general microbiology, biofilm formation and key characteristics, and medical interventions of biofilm infections including treatment options.

There are several factors that we consider important in this activity. The first is increasing science capital [15, 16]. This was achieved by developing the activity using inexpensive materials, and using a simplistic, minimalistic approach to communicate somewhat complex concepts. Making the activity accessible to all was an important consideration during development, and using building blocks was a perfect approach because if participants want to continue exploring biofilm development, building blocks can be found in most households, or in many public establishments, readily available. Asking questions to prompt discussion points gave the participants an opportunity to think and engage with considered responses, where an encouraging environment made them feel integral to the activity. Although it is generally more difficult to engage with introverted or reserved participants, we found that the common, relatable materials used in this activity, along with the fact that many children enjoy playing with plastic building blocks to express themselves in any creative way they wish, allowed everyone to engage and participate. Although there are certain rules (to ensure the activity is manageable), participants were free to design and build any structure they wish. We found that this allowed their creativity and teamwork skills to flourish. There were natural leaders within each group, but participants communicated well with each other, contributing to skill development. Although the activity is relatively short in time, dexterity and concentration are also important skills that are utilized and through repeated delivery, can continue to develop.

Accessibility of STEM education and careers begins at home [16], and our primary target audience includes participants from either geographic or socio-economical areas that do not typically associate with STEM. Many of the participants we deliver activities to are would-be first-generation university attendees, and so there is no relatability for STEM careers. Through numerous discussions with the participants, they often describe feeling that it is an unachievable goal, whether for academic or social reasons, or that the perceived stereotype of a scientist rings true, and they feel they would not belong in a STEM career. Through activities such as these, and ensuring we actively demonstrate and encourage diversity, we can enhance the feeling of ‘worth’, and demonstrate that there are a huge range of STEM careers that often go undervalued or opportunities that are simply not communicated well enough [17]. This also contributes to the sense of identity status and identity capital [18].

We have demonstrated that science communication can be as minimalistic and as simple or indeed as complex as you want it to be, and through co-development of activities with teachers and other participants, and continued adaptation and development based on a robust evaluation and feedback system, it is possible to inspire participants that may not otherwise have any interest in pursuing a STEM career. This activity contributes to science capital, a sense of belonging and worth, and has short and longer-term impact for participants, as well as contributing to the development and scientific knowledge of teaching professionals, for a long-lasting effect.

## Community resources

Open access resources related to this activity are available for delivery with credit to Show Me The Science. They can be accessed at the following website address: https://www.showmethescience.co.uk/biofilms

Please contact Show Me The Science before modifying these resources.

Risk assessments should be completed prior to the public engagement activity (a template of which is available as part of the resources above). Take into consideration the age and ability of the participants, and the physical aspects of trying to disrupt/destroy the biofilm structure. This involves throwing objects at the biofilm. Personal protective equipment including safety glasses and exclusion areas may be appropriate. An example risk assessment is also provided as part of the community resources.

Posters and additional information for the activity are available in the Supplementary Materials.

## Summary/conclusion

This activity highlights several key areas in clinical and environmental microbiology, specifically biofilms, their development and structure; and from a clinical perspective, the inherent increased resistance to treatment. The structure of the activity (introducing the theory, demonstrating, allowing participants to engage, then reviewing) has been very well received by children (Key Stage 2) and teaching staff, and participants of all ages who experienced this activity during public events. There is no requirement for specialized equipment, and the materials are basic and generally low in cost. The inclusion of a workshop handout detailing the materials and methods also allows for redelivery either in schools or at home.

## Peer review history

### VERSION 2

#### Editor recommendation and comments

https://doi.org/10.1099/acmi.0.000467.v2.3

© 2022 Efthimiou G. This is an open access peer review report distributed under the terms of the Creative Commons Attribution License.

**Georgios Efthimiou**; University of Hull, Biomedical Science, Hardy Building, Cottingham Road, UNITED KINGDOM, Hull

Date report received: 07 November 2022

Recommendation: Accept

**Comments**: The work presented is clear and the arguments well formed. This study would be a valuable contribution to the existing literature. This is a study that would be of interest to the field and community. All comments by the reviewers were satisfactorily addressed.

#### SciScore report

https://doi.org/10.1099/acmi.0.000467.v2.1

© 2022 The Authors. This is an open-access article report distributed under the terms of the Creative Commons License.

#### iThenticate report

https://doi.org/10.1099/acmi.0.000467.v2.2

© 2022 The Authors. This is an open-access article report distributed under the terms of the Creative Commons License.

#### Author response to reviewers to Version 1

Author response to reviewers’ comments

Firstly, I would like to thank the reviewers for the time taken to review the manuscript and offering suggestions in ways we can improve.

We have carefully considered the recommended changes and have detailed these changes in the individual comments below. We hope that this is sufficient for acceptance for publication.

**Editor:**

1. In the Introduction - increase public engagement, why do we need to tell children about science? And biofilms?

Thanks for this comment – we have updated the introduction substantially to include more about outreach, engagement, and why biofilms.

2. Give few more details about the participants.

We have updated this in the manuscript (L161-168)

3. Reduce the size of the Required materials section.

This has been removed and incorporated into the practical activity section. This has been adapted to past tense (as with typical scientific articles)

5. In Evaluation - please include any feedback from the participants if available.

Have included some data based on recent school visits – see Data Collection and Evaluation section (Line 254 onwards)

6. In Conclusion - increase discussion, add 10-15 more references.

We have separated the discussion and summary/conclusion, increasing the number and breadth of citations. We feel this discussion should be sufficient for the type of manuscript.

**Reviewer 1 Comments to Author:**

Thank you for taking the time to write this manuscript, it's really important for the microbiology community to hear about outreach activities.

The activity described in this manuscript is relevant to both the scientific community and school children and so is a lovely example of public engagement.

I feel the manuscript would be better written from the perspective of 'this is what we did', rather than a 'pick off the self activities'.

Thank you so much for the kind words – we agree, microbiology is an often underappreciated concept in outreach/public engagement, and through activities such as these, we can bring this to the forefront of the general public!

The original perspective of the manuscript, as an educational article, was for provision of this activity (a ‘this is what you can do’ rather than a typical ‘this is what we did’ approach). We have considered this comment carefully, and have restructured the manuscript to follow more typical research-based manuscripts.

Major comments

Introduction - increase public engagement, why do we need to tell children about science? And biofilms? (could link to science capital here - see Archer papers), what has already been done. I think the intro should be 40% biofilm 60% public engagement.

Indeed, science capital is an important aspect we haven’t gone into much detail of in this manuscript. We are very aware of Archer’s work, and have updated the introduction to reflect more of a pedagogical approach for this as recommended.

Participants- after the introduce please include a section on the country, age, setting and number of participants. Also the average group size for the activity etc, so reader can get a picture of what you sessions looked like in terms of the participants

We have updated the section with this information. We intentionally left this out as it wasn’t intended as a retrospective analysis of a specific study, but a set of instructions for outreach provision where audiences will of course differ depending on geography and target audience. However, we have changed the approach of the manuscript, and have updated this section to reflect this.

Required materials - I feel this section is too long and given too much space in the paper, condense and rewrite as past tense (what you did) and merge with the next section

We have removed this and incorporated it into the practical activity section.

Practical activity - rewrite as past tense and focus on what you did, it might be useful to have subheading for each of the different activities. Please include figures in the section (fig 1 and 2 are great), some of the supplementary material could be used as figures in this section.

We have re-written this section to accommodate a number of suggestions from reviewers. Figures 1 and 2 have been moved into this section. We feel this is sufficient for the main manuscript and so have not moved any supplemental information to the main manuscript.

Evaluation - You have not done any detailed evaluation of the activity which is a real shame as a section after the practical on feedback would really benefit the paper and the discussion. Could you put in some ethics to able to ask the teachers what they thought of the activity and then you could analysis and include some quotes? Or do you already have some you could include (this is hinted at)?

Thank you for this observation. We intended the original submission to be a guide for provision rather than a study of the outreach project. As detailed above, we have changed this approach and some data has been incorporated into this updated manuscript.

Community resources - write a section before the conclusion entitled "community resources" - here you can include links to the resources you have made in the resources and frame it as open access resources. Are people ok to use and adapt these as they see fit? Also include in this section the safety paragraph.

We are happy for some resources to be publicly available assuming credit is given to the outreach group, as an independent business identity. We have provided a link to the website where the resources can be found. We require formal requests to adapt the activity or resources prior to delivery.

Conclusion - increase discussion, include an overview of what the resource is and how it supports public engagement.

This has been increased

References - increase links to public engagement literature - I would like to see 10 -20 references please

The list of references has been enhanced

Minor comments

Key words: public engagement (rather than treatment)

Updated, thanks for this suggestion.

**Reviewer 2 Comments to Author:**

I like this as it seems a very accessible way to teach children about microbiology and biofilms. I do have a couple of specific comments that are listed below.

Thank you for the kind words!

line 101 - ping pong --> table tennis.

Have updated this phrasing in the text and supplementary file

line 147 - it is stated that the educator can introduce the concept of persister/resistant cells, but that concept is not explained in appendix 1. This appendix needs a section on resistance and antimicrobials, and it needs to be clarified why different types of balls can be used in the exercise.

Thanks for this comment. We have updated this in the text of Appendix 1.

Appendix 1 - there is some terminology that not every educator may be familiar with, such as van der Waals forces (line 59).

We have elaborated on this in the text.

On line 105, the terms facultative and fastidious do not seem necessary.

Thank you, this has been removed

Line 120. Typo: alto --> also

Sorry for this mistake! Thanks for bringing it to our attention, amended.

Figure S2 - It would be helpful to add some labelled arrows to the image, with an explanation in the legend, so that this is clear for the educator what they are looking at. It may also not be obvious to everyone that this is polymicrobial - use arrows top point out eg rods and cocci.

Thanks for this comment. This figures have been updated accordingly.

On the poster, individual microbes in panel C are not obvious, a different image would be better.

The image has been updated.

### VERSION 1

#### Editor recommendation and comments

https://doi.org/10.1099/acmi.0.000467.v1.5

© 2022 Efthimiou G. This is an open access peer review report distributed under the terms of the Creative Commons Attribution License.

**Georgios Efthimiou**; University of Hull, Biomedical Science, Hardy Building, Cottingham Road, UNITED KINGDOM, Hull

Date report received: 23 August 2022

Recommendation: Major Revision

**Comments**: The work presented is clear and the arguments well formed. This study would be a valuable contribution to the existing literature. This is a study that would be of interest to the field and community. The reviewers have highlighted major concerns with the work presented. Please ensure that you address their comments. This manuscript is interesting and well-written, a great example of innovative public engagement. Few major changes are needed before acceptance: 1. In the Introduction - increase public engagement, why do we need to tell children about science? And biofilms? 2. Give few more details about the participants. 3. Reduce the size of the Required materials section. 5. In Evaluation - please include any feedback from the participants if available. 6. In Conclusion - increase discussion, add 10-15 more references.

#### Reviewer 1 recommendation and comments

https://doi.org/10.1099/acmi.0.000467.v1.3

© 2022 Lacey M. This is an open access peer review report distributed under the terms of the Creative Commons Attribution License.

**Melissa Lacey**; Sheffield Hallam University, Department of Biosceinces and Chemistry, Howard Street, Sheffield, UNITED KINGDOM

https://orcid.org/0000-0003-0997-0217

Date report received: 16 August 2022

Recommendation: Major Revision

**Comments**: Thank you for taking the time to write this manuscript, it's really important for the microbiology community to hear about outreach activities. The activity described in this manuscript is relevant to both the scientific community and school children and so is a lovely example of public engagement. I feel the manuscript would be better written from the perspective of 'this is what we did', rather than a 'pick off the self activities'. Major comments Introduction - increase public engagement, why do we need to tell children about science? And biofilms? (could link to science capital here - see Archer papers), what has already been done. I think the intro should be 40% biofilm 60% public engagement. Participants- after the introduce please include a section on the country, age, setting and number of participants. Also the average group size for the activity etc, so reader can get a picture of what you sessions looked like in terms of the participants Required materials - I feel this section is too long and given too much space in the paper, condense and rewrite as past tense (what you did) and merge with the next section Practical activity - rewrite as past tense and focus on what you did, it might be useful to have subheading for each of the different activities. Please include figures in the section (fig 1 and 2 are great), some of the supplementary material could be used as figures in this section. Evaluation - You have not done any detailed evaluation of the activity which is a real shame as a section after the practical on feedback would really benefit the paper and the discussion. Could you put in some ethics to able to ask the teachers what they thought of the activity and then you could analysis and include some quotes? Or do you already have some you could include (this is hinted at)? Community resources - write a section before the conclusion entitled "community resources" - here you can include links to the resources you have made in the resources and frame it as open access resources. Are people ok to use and adapt these as they see fit? Also include in this section the safety paragraph. Conclusion - increase discussion, include an overview of what the resource is and how it supports public engagement. References - increase links to public engagement literature - I would like to see 10 -20 references please Minor comments Key words: public engagement (rather than treatment)

*Please rate the manuscript for methodological rigour*

Satisfactory

*Please rate the quality of the presentation and structure of the manuscript*

Poor

*To what extent are the conclusions supported by the data?*

Partially support

*Do you have any concerns of possible image manipulation, plagiarism or any other unethical practices?*

No

*Is there a potential financial or other conflict of interest between yourself and the author(s)?*

No

*If this manuscript involves human and/or animal work, have the subjects been treated in an ethical manner and the authors complied with the appropriate guidelines?*

Yes

#### Reviewer 2 recommendation and comments

https://doi.org/10.1099/acmi.0.000467.v1.4

© 2022 Bolhuis A. This is an open access peer review report distributed under the terms of the Creative Commons Attribution License.

**Albert Bolhuis**; University of Bath, Pharmacy and Pharmacology, University of Bath, Dept. of Pharmacy and Pharmacology, Claverton Down, Bath, UNITED KINGDOM

https://orcid.org/0000-0001-9307-0515

Date report received: 19 August 2022

Recommendation: Minor Amendment

**Comments**: I like this as it seems a very accessible way to teach children about microbiology and biofilms. I do have a couple of specific comments that are listed below. line 101 - ping pong --> table tennis. line 147 - it is stated that the educator can introduce the concept of persister/resistant cells, but that concept is not explained in appendix 1. This appendix needs a section on resistance and antimicrobials, and it needs to be clarified why different types of balls can be used in the exercise. Appendix 1 - there is some terminology that not every educator may be familiar with, such as van der Waals forces (line 59). On line 105, the terms facultative and fastidious do not seem necessary. Line 120. Typo: alto --> also Figure S2 - It would be helpful to add some labelled arrows to the image, with an explanation in the legend, so that this is clear for the educator what they are looking at. It may also not be obvious to everyone that this is polymicrobial - use arrows top point out eg rods and cocci. On the poster, individual microbes in panel C are not obvious, a different image would be better.

*Please rate the manuscript for methodological rigour*

Good

*Please rate the quality of the presentation and structure of the manuscript*

Good

*To what extent are the conclusions supported by the data?*

Strongly support

*Do you have any concerns of possible image manipulation, plagiarism or any other unethical practices?*

No

*Is there a potential financial or other conflict of interest between yourself and the author(s)?*

No

*If this manuscript involves human and/or animal work, have the subjects been treated in an ethical manner and the authors complied with the appropriate guidelines?*

Yes

#### SciScore report

https://doi.org/10.1099/acmi.0.000467.v1.1

© 2022 The Authors. This is an open-access article report distributed under the terms of the Creative Commons License.

#### iThenticate report

https://doi.org/10.1099/acmi.0.000467.v1.2

© 2022 The Authors. This is an open-access article report distributed under the terms of the Creative Commons License.

## Supplementary Data

Supplementary material 1
