# Supplementary material for: Building blocks of biofilms – an engaging and hands-on microbiology outreach activity for school children and the general public
Source: Access Microbiol. 2023 Feb 3;5(2):000467.v3. doi: 10.1099/acmi.0.000467.v3 (PMC9996183; doi:10.1099/acmi.0.000467.v3)

Supplementary material for Building Blocks of Biofilms

Building Blocks of Biofilms – an engaging and hands-on microbiology outreach activity for school children and the general public

Running title: building blocks of biofilms

Hayley Pincott[1,2], Megan Hughes[1], Thomas Cummins[1], **Daniel J Morse[1]**

1 Show Me The Science, Cwmbran, UK

2 Oral Pathology & Microbiology, University Dental Hospital, Cardiff, UK

Corresponding Author: Daniel J Morse, daniel@showmethescience.co.uk

Table of contents:

Appendix 1. Detailed background information for educators

Appendix 2. Detailed instructions for practical activities

Appendix 3. Instructional poster for practical activities

Appendix 4. Biofilms educational poster

Appendix 1. Detailed background information for educators

Introduction.

Biofilms are defined as aggregates of microorganisms (frequently as more than one type living together; termed polymicrobial), often attached to a surface and embedded in a matrix of self-produced polymeric substances. Their distribution in the natural environment is ubiquitous. They can exist on natural or man-made abiotic (non-living) surfaces such as rocks, floors, walls etc., or on biotic (living) surfaces, including wounds, teeth or skin/mucosal surfaces. Importantly, they can exist at the interface of both abiotic and biotic surfaces, and in healthcare they are a significant burden both to the patient and the health service resources when they colonise and infect a prosthesis such as an implant or replacement joint.

Approximately 60% of healthcare-associated infections are thought to have a biofilm origin, and they can have some very substantial implications in our general health, particularly with oral hygiene. In order to explain and discuss the concept of biofilms, we will be using the familiar example of dental plaque as a familiar reference. After a period of restricted or poor oral hygiene, a plaque film begins to develop on the tooth surface. This is the archetypal example of a biofilm – the structured community of many different microbial species, attached to the tooth surface, and embedded in the self-produced outer matrix, which follows the defined rules and sequence of development.

Biofilm Formation

The pellicle (surface coating)

Hundreds of different types of microorganisms exist in the mouth, and they can be found suspended in their free-living state in saliva, known as the 'planktonic' state. Contrary to what was previously thought, we now understand that the planktonic method of growth is not the preferred way for most microorganisms to grow, but they tend to form biofilms. This is important for their survival, particularly in the oral cavity where there is an ongoing risk of them washed away by the continual movement and swallowing of saliva.

Biotic (living) surfaces are rarely free of microbial matter. This is particularly true in the oral cavity, where the saliva provides a coating (pellicle) on the surfaces of the teeth and gums. This pellicle contains a myriad of proteins and sugars, and makes it much easier for microorganisms to attach, as there are far more receptors for them to bind to. Microorganisms have adapted over thousands (even millions) of years to have the ability to attach to a range of surfaces and survive, and they do this through very refined mechanisms, including forming biofilms which follow a distinct sequence of events for formation detailed below. This pellicle not only supports a physical attachment, but the proteins and sugars can also be used as a source of nutrients for the primary microbial cells that exist there.

Stages of biofilm development; attachment/adherence

Figure S1 details the stages of biofilm formation. Planktonic microbial cells are continually moving around suspended in the saliva, and where they come into close proximity to the surface, weak attractive forces come into play. Forces such as Van der Waals, electrostatic or ionic forces attract the microbial cell toward the surface (Fig. S1a), where it can stick and eventually attach to specific receptors. Van der Waals forces are a distance-dependent attraction/repulsion of two objects, based on interactions between atoms or molecules. Similarly, electrostatic forces are non-contact forces but rely on differences in charges between two objects to be attracted (to push away or pull towards each other). For example, a positively charged object is attracted to a negatively charged object, but will be ‘pushed away’ or repelled from another positively charged object. Microbial cells have charges on their outer surface, and this can influence whether they are attracted to or repelled from the surface. However, the surface itself also has a charge, and depending on what is present on the surface can dictate the charge this has, and therefore whether it attracts or repels other objects. These early attractive forces between the microbe and the surface are relatively weak, so if there is, for example in the oral cavity, a strong salivary flow, these microbial cells can become detached from the surface and remain in their planktonic phase. However, shortly (approximately 60 minutes) after attachment, and in the absence of stronger forces, the cells more strongly adhere to the surface using locking receptor interactions. This is the first stage of biofilm formation.

The stronger binding mechanism of locking receptor interactions work similar to a hook and loop concept. The microbial cell has proteins (ligands) on the outer surface known as adhesins, that stick out looking for a suitable receptor to bind to. When it comes across something suitable, it will ‘hook on’ to the receptor, making a much stronger binding/connection, which is deemed irreversible and not subject to being washed away with the same forces as detailed above. Different microbes have different adhesins that stick to different receptors, but an awareness of the hook and loop mechanism is sufficient here.

Stages of biofilm development: colonisation

The first microbial cells to attach to the surface are often referred to as ‘primary colonisers’ – the first to colonise the surface. These are typically those in highest abundance in the environment and those that can exist in a range of environmental conditions. For example, *Streptococcus* bacteria are often found during the early formation of dental plaque, and are known to be some of the first colonisers of many oral surfaces. Having the primary colonisers attached to a surface now makes it much easier for the eventual formation of a biofilm. Additional planktonic microbial cells not only need to attach to the tooth surface with specific proteins to bind to, but have the opportunity to bind to other cells that have already adhered to the surface (Fig. S1b). These cells are called ‘secondary colonisers’. Many microorganisms can interact with each other, either by producing chemical signals that they secrete into the environment, or by direct contact. This direct contact interaction is similar to the primary coloniser interacting with the surface and the bond is considered to be relatively strong, and is known as co-aggregation. Free-floating biofilms (floccules) also exist, and these are similar to surface-associated biofilms, as they consist of co-aggregated cells and are often embedded in a matrix, but are not attached to a specific surface. These have been found in environments such as streams and rivers, where the water flow is too high for them to attach to the static surfaces, but they still maintain the ability to cluster together in a biofilm form.

The secondary colonisers may attach directly to the primary colonisers, resulting in a second layer of cells still attached to the surface. This layering continues with more and more microbial cells attaching

to the outermost layer, and as a result of cell multiplication from within the biofilm, until a multi-layered structure is formed (Fig. S1c). This is the typical structure of a biofilm. A high-magnification microscopy image of a polymicrobial biofilm grown under laboratory conditions illustrating this structure is shown in Fig. S2.

Stages of biofilm development: growth/maturation and production of the important outer matrix

As the biofilm structure establishes and grows into this 3D structure, many of the cells produce substances that contribute to building a protective matrix (Figs. S1c-d), such as secreted proteins, sugars and DNA from the cells and environment. This provides a physical structure for the biofilm onto which other cells can attach and establish themselves, further encouraging growth.

The matrix has multiple purposes. It works as a physical scaffold for continued biofilm growth and maturation into a 3D structure meaning other planktonic microorganisms can join the biofilm structure. It also provides physical protection from environmental fluctuations or extremes such as changes in pH, temperature, nutrient availability, and acts as a physical barrier to treatments by antimicrobial compounds. As biofilms are primarily made up of water, and rely on that for the survival of the contained microorganisms, the matrix acts to stop desiccation in warmer and drier environments.

The biofilm community works together for the greater good of survival, and there are various ways in which the microorganisms can help each other for this to be achieved. Many microorganisms require oxygen to survive (aerobic), but there are also many microorganisms that cannot survive in the presence of oxygen (anaerobic). However, both of these are able to exist in the same biofilm in what seems to be the same environmental conditions. This is possible through the formation of oxygen micro-gradients. Bacteria reliant upon oxygen consume the oxygen that is present, forming oxygen-limited environments which then supports the life of those that require the absence or low levels of oxygen. This symbiotic (mutually beneficial) relationship is one example of many similar relationships where some microorganisms need certain nutrients or conditions that can only be produced by others in that

community. In return, those that benefit from changes may produce factors that break down antimicrobials or change different environmental conditions such as acidity.

Stages of biofilm development: dispersal

Within this protective biofilm environment, housed by the robust matrix, the biofilm cells continue to develop into the 3D structure, whilst continuing to produce the matrix through to a mature biofilm state. The length of time this takes varies depending on the type of microorganisms that are growing. Dental plaque can establish a relatively mature biofilm within a couple of days. However, environmental biofilms may take weeks as they all require different nutrients and conditions to support their growth, despite being highly adapted to their surroundings. Anaerobic microorganisms also tend to take a lot longer to grow, as they are far slower in terms of growth than aerobic counterparts.

Scientists understand that dispersal actually happens both actively (e.g. intentionally) as a result of environmental stresses, antimicrobials, environment conditions etc, and passively as a result of too high a water flow where the cells are sloughed off the main biofilm. Because the cells within the biofilm are generally attached to each other relatively strongly, it requires an enzyme to break the bonds between the cells and to detach from the sticky matrix in order for it to be able to break free. Sometimes, biofilms that exist on the gums can enter the bloodstream if there is localised damage to the gum, and this is an easy way for the biofilm cells to get into the heart and valves where some microorganisms can cause endocarditis (an infection of the inner heart). The easiest example of passive dispersal would be of biofilms in water pipes, or rivers and streams.

Persister cells, antimicrobial resistance and use of antimicrobial balls in this activity

During treatment and/or removal of biofilms from any surface, it is almost practically impossible to remove every single microbial cell. This happens for a myriad of reasons, some of which we will discuss here.

Firstly, a biofilm is a complex community of microorganisms, many of which will have an inherent ability to resist, withstand or tolerate antimicrobial treatment by chemicals, medication, or drugs. This is due to several mechanisms in the cells, such as producing enzymes to break down the drugs, pumps to actively pump the drug/chemicals outside of the microbial cell if they get in, a thick matrix surrounding the biofilm itself to withstand penetration, differing pH within the biofilm structure, and reduced oxygen concentration inside the matrix. All of these affect how the drug works and whether it will be able to kill the cells or destroy/remove the biofilm structure.

The microorganisms that are closest to the surface or in the middle of the 3D biofilm structure will also undergo a process to reduce their metabolic activity, effectively going into a state of hibernation. Many drugs work by the metabolism or growth of microorganisms, and so simply not growing or multiplying is a great way to avoid being targeted by the drug. These are known as **persister cells**, and can stay in this state of hibernation for a long time, beyond typical treatment periods. Then, when the conditions in their local environment change to favour their growth, they will do so and have the ability, in the absence of the drugs/chemicals, to grow and re-form a second biofilm, where the process continues.

This activity aims to introduce the concept of antimicrobials as medications and treatments for infections, through the use of soft balls. The balls are used by the participants to destroy the biofilm structure, but those that stick to directly to the surface of the building block plate tend to do so very strongly, and are not removed by these 'medicine balls'. These are the persister cells, which the antimicrobial balls cannot remove (for reasons that can be discussed as detailed above, allowing the participants to suggest or think about why that is). Additionally, introducing the concept of natural acquisition of resistance can be done here. The generation time of microorganisms can be as short as 20 minutes, and with many new generations, come natural mutations in the DNA sequence of the prodigy. Some of these mutations will be beneficial, and may infer a trait such as resistance to a drug. Then, when that particular drug is used to treat the biofilm, these inherently resistant microorganisms will remain viable after the treatment, and then continue to grow and multiply, meaning the newly formed structure will also be resistant to the previously used drug that wiped out the last community

(after inheriting the same DNA as their 'parent cells'). This makes it much more difficult to treat subsequent biofilms because there are fewer options of drugs that will be effective.

Introducing different types of balls (soft snow balls versus table tennis balls, for example) is a way to demonstrate selecting the correct drug for the type of infection. If the participants build a biofilm structure, refer to that as a fungal biofilm. The soft snow balls are 'antifungal drugs', whereas the table tennis balls are antibacterial nanodrugs. When the soft snow balls are used, they destroy the structure relatively easily, because the drug is an antifungal targeting a fungal biofilm. However, the antibacterial nanodrugs (table tennis balls) are targeting bacterial cells, where there are none, and therefore have limited to no effect on the biofilm structure. This emphasises the selection of the right drug to treat the right type of infection.

It is important in this activity to complete the treatment steps in stages, which can be referred to as 'doses'. Each participant receives one 'antimicrobial ball', and they throw that at the structure. This is the first dose. After this, the structural integrity is reviewed, and it will likely not have destroyed very much. Then the participants complete a 'second dose', and then another review, and then a third dose and so on. Each 'dose' will destroy more of the biofilm structure, which also relates to real-life situations where several doses of a medication is required to have a sustained, cumulative effect.

Not all bad news

Whilst we do tend to think of biofilms as being the bad guys of medicine, they deserve to be seen in a negative light when they are healthcare-associated, but biofilms are also very useful in our daily lives. In fact, we have biofilms that live in our gut and help to break down foods; they are used in water and wastewater treatment plants as a pre-treatment to feed on dead tissues or cells; and are involved in bioremediation or waste breakdown. They are even used in the production of alcohol through fermentation processes! So while there may be a lot of generalised negativity towards 'germs' and biofilms, it is important to highlight the benefits of controlled biofilm use,.

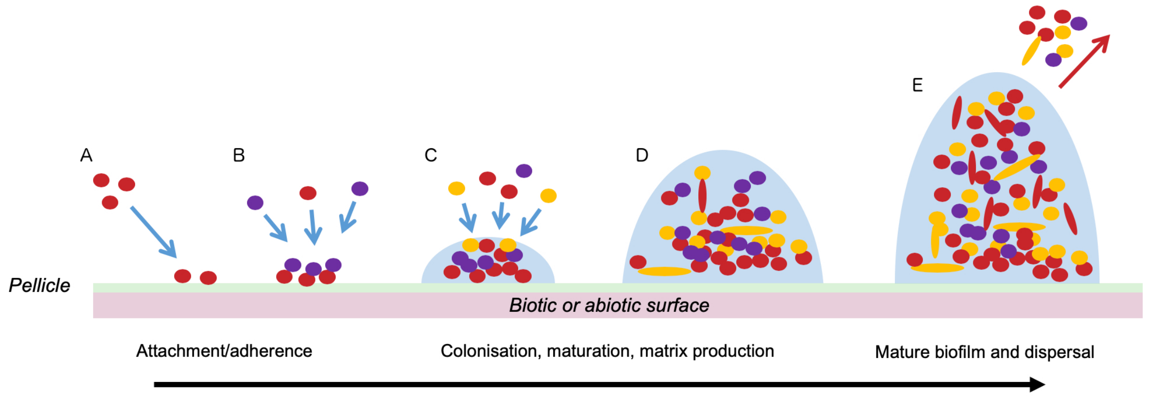

Figure S1. The process of biofilm formation in defined stages; attachment to the surface pellicle, colonisation and maturation, and dispersal. **A)** Attachment to the surface from planktonic phase, **B)** secondary-colonisers co-aggregating, **C)** colonisation, continued recruitment of microorganisms and replication from within, and matrix production, **D)** begin maturing in 3D manner, continued matrix production and **E)** dispersal of cells.

Image modified with permission from Dr D Morse PhD thesis.

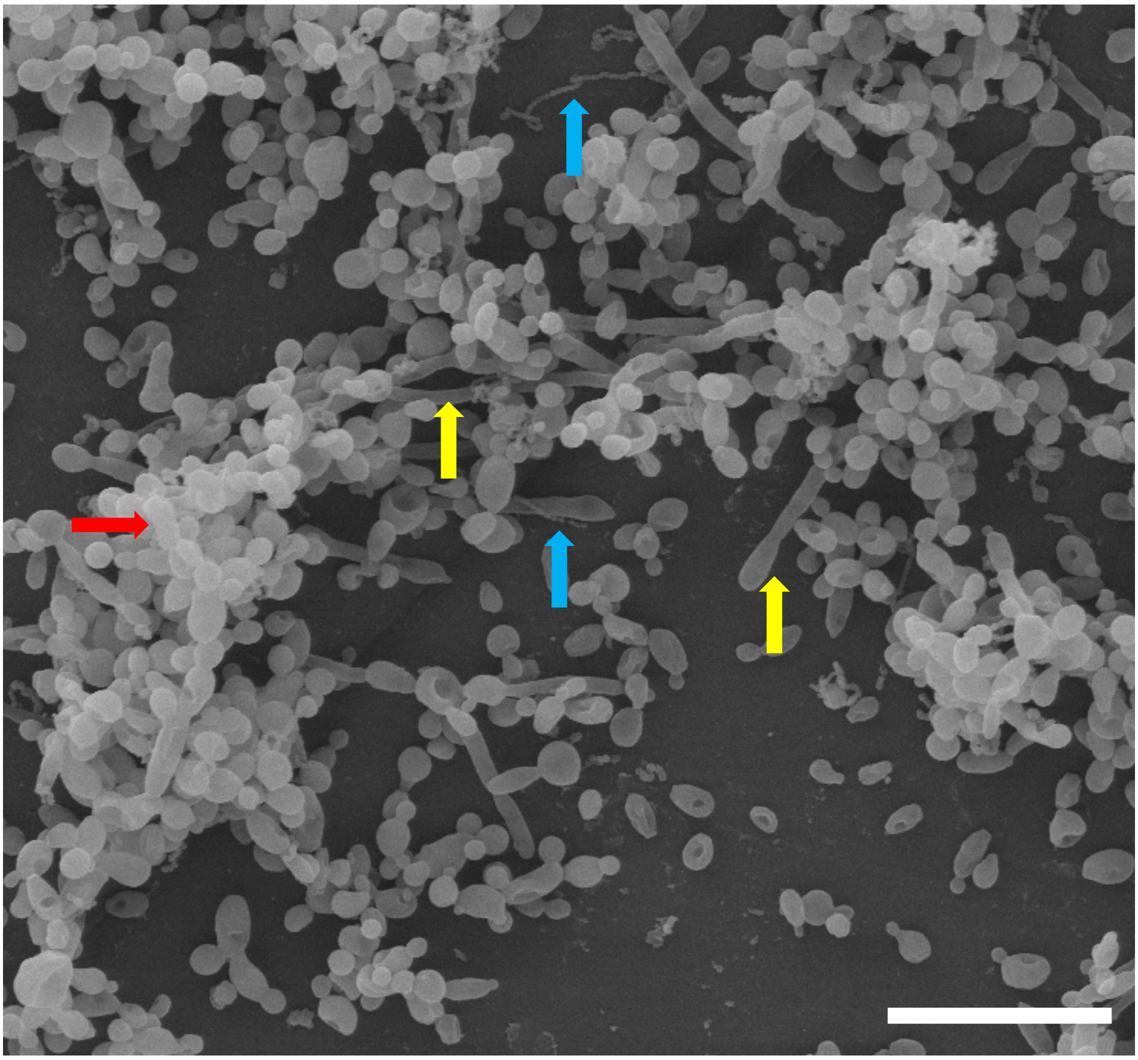

Figure S2. High magnification scanning electron microscopy image of polymicrobial biofilm grown on denture acrylic material. The typical multi-layered structure of the biofilm is clearly visible, with cells co-aggregating into a cluster attached to the surface. Smaller colonies at the earlier stage of biofilm development are also visible. The white bar represents 20 µm in length (0.02mm). The red arrow shows the larger cells of the fungus *Candida albicans* (about 10x larger than bacteria). The yellow arrows show the different shape that *C. albicans* can take on (hyphae – elongated shape). The light blue arrows show the much smaller cocci-shaped bacteria cells arranged in chains.

Appendix 2. Detailed instructions for practical activities

1. Dental plaque disclosing tablets

Required materials:

- Drinking water
- Recyclable cups (plus one for waste)
- Disclosing tablets (1 per person)

Method:

- Insert disclosing tablet into mouth and chew whilst mixing around in saliva and washing around the mouth for approximately 30 seconds.
- Sip a small volume of water and rinse around mouth, then spit into waste container.
- Look at colouration of plaques on teeth and gum line.
- Pink colour indicates new plaque, blue/purple colour indicates older plaque.

Discussion points:

Dental plaque formation (e.g. biofilm), position of plaques on teeth, spread of plaques around front/back teeth and quantity. Brushing frequency, techniques and duration, and the need to brush with fluoride toothpaste (to reduce plaque bioburden and maintain enamel integrity).

2. Building Biofilms

Required materials:

- Large plastic interlocking building blocks (of varying shapes and sizes). A quantity of 50-100 per team is ideal.
- Building block base plates suitable for the blocks (2x)
- Soft balls for destroying the biofilm structure (examples include plastic soft-play balls, soft cotton ‘snow balls’, table tennis balls etc. Dense but soft balls such as the snow balls are preferred and have been evaluated for suitability.

Method:

- Begin by marking out and explaining the area in which the participants will be building their biofilms. Approximately 10 x 10 pegs is normally suitable for this activity.
- Explain briefly the typical process of biofilm formation to the participants, using blocks to act as different microbes (and the Methods Poster (Appendix 4) as a reference):
    - Start with freely floating primary colonisers that stick to the surface. Attach a number of these to the pegs.
    - Secondary colonisers then bind to the already attached primary colonisers. It is important to emphasise that the microbes never typically bind covering the whole surface (in this case avoiding complete coverage of the blocks), but they attach to the edges/outermost block surfaces. For example, on a 2x2 block, another block would cover 1x2 pegs, leaving the others free. The next block would then either attach to the remaining 1x2 pegs, or similarly, 1x2 on the second layer block. See image below.
    - It is also necessary to emphasise the importance of channels within the biofilms. These channels are essential for nutrient and gaseous transfer into and within the biofilm structure, and therefore needs to be represented in this biofilm structure. The participants are not to build a tower-like structure, with impenetrable outer 'walls', but to allow channels for these nutrients to pass in and around, to support the life and growth of microbes deep within the biofilm depths and closest to the surface.
- Split the participants into two teams, each with a board and a set quantity of building blocks. If there are many participants, consider sub-splitting these into different groups for the following approach.
- Allow the participants to build the biofilms in a given time period (e.g. 1-2 minutes, but to be guided by the speed in which they are building successfully). If teams are too large, and need to be split into smaller groups within the teams, assign these a number or letter (e.g. Team 1 group A, Team 1 Group B etc.). Allow 30-60 seconds for each group to build the biofilm structure, after which the groups switch places, and the next group continues to build on the previous structure. This is continued until each group has contributed to the structure, and/or

the overall time allowance has elapsed. Any unused blocks are counted but removed from the area.

- The biofilm structure is quantified by number of blocks used (or by subtraction of those not used), and then the educator can discuss the structure of the biofilms. Highlight the typical characteristics of the biofilm such as shape, height and presence of channels.
- The teams switch to the other team's biofilm, and are given the antimicrobial balls in preparation for destroying the structure. This is to be gauged on number of participants, but ideally 1-3 attempts to destroy per participant is normally sufficient.
- Allow the participants to throw the balls against the biofilm structure, and destroy as much as they can before switching to the other team and other biofilm structure. Count the number of blocks (microbes) that remain attached to the surface, and calculate percentage removal of biofilm mass. The team that removes the highest percentage of biofilm structure is considered the winner.
- This can be repeated with alternative 'antimicrobial balls', or if discussed prior to the activity, the participants can choose their favoured 'antimicrobial ball' from an available range for the type of infection the educator wants to portray.

Appendix 3. Biofilms educational poster

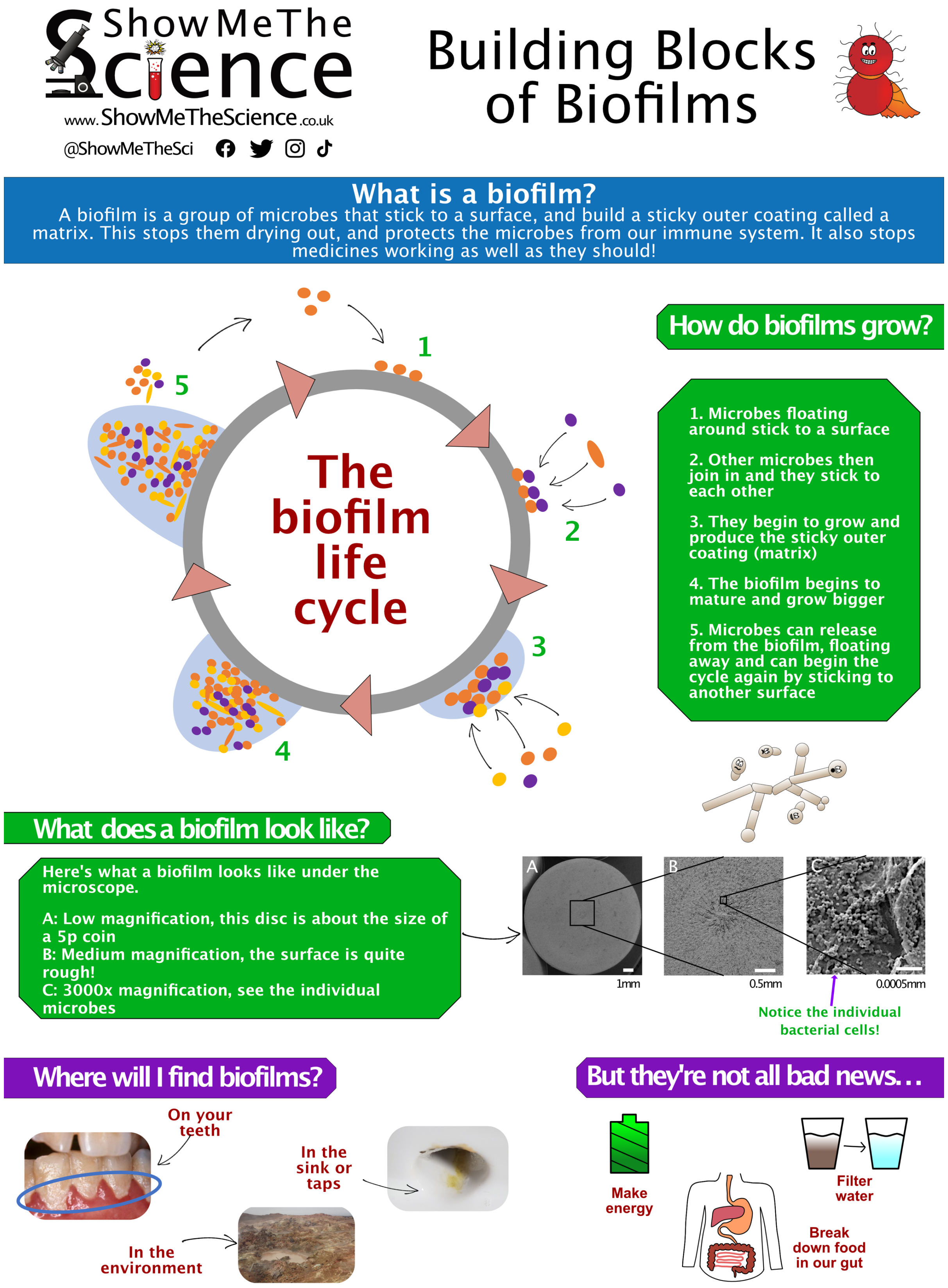

Appendix 4. Instructional poster for practical activities

ShowMeThe Science
www.ShowMeTheScience.co.uk
@ShowMeTheSci

Building Blocks of Biofilms

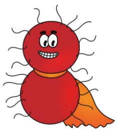

Let's get building biofilms!

Using the instructions listed below, and the information you've already been told, build a biofilm!

Rules:
1. Stay within the lines. Blocks can overhang outside of this area.
2. Allow spaces for oxygen/nutrients for the microbes - they can't survive if they're built like a tower!
3. Microbe blocks stick together on the edges, not flat together!
4. HAVE FUN!

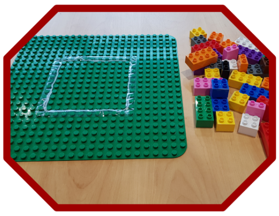

1. Microbes come in all shapes and sizes

Use as many blocks in any order you want, but stick to the rules or you may have to start again!

Why not make it a competition between you and a friend or family member? Who can build the best/strongest biofilm?

2. Microbes free-floating in the environment stick to the surface, much like these blocks stick to the plate! This happens over and over again until there are lots of microbes on the surface - but, they always keep some gaps so they can get oxygen and nutrients!

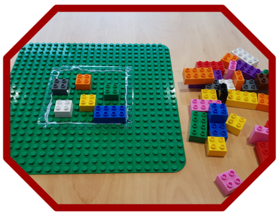

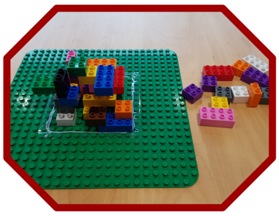

3. Next, more microbes will join and stick to each other and begin to build up in a tower. Remember, they normally stick to each other on the edges, and have gaps for their nutrients and oxygen.

4. The biofilm continues to grow upwards and outwards, into a big tower like structure with gaps and channels so the ones at the bottom can still get the nutrients they need.

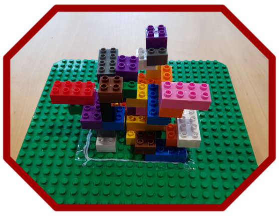

How strong is your biofilm!?

Time to use our 'antimicrobial balls' and destroy the biofilm!

How much did the different medicines destroy?
What happened at the end? Did you manage to get rid of all the microbes?

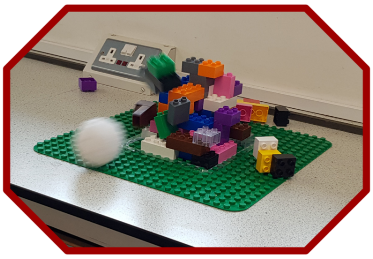

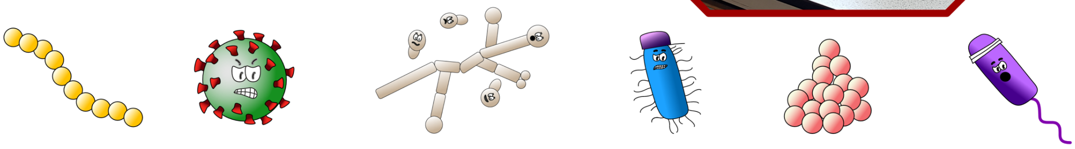

Supplement: Supplementary material 1 [file acmi-5-467.v3-s001.pdf]
